# Intravenous tocilizumab for the treatment of giant cell arteritis: a phase Ib dose-ranging pharmacokinetic bridging study

**DOI:** 10.1186/s13075-022-02815-9

**Published:** 2022-06-04

**Authors:** Christophe Schmitt, Laura Brockwell, Mylène Giraudon, Mauro Zucchetto, Lisa Christ, Bettina Bannert, Thomas Daikeler, Peter M. Villiger

**Affiliations:** 1 Department of Clinical Pharmacology, F. Hoffmann-La Roche AG, Grenzacherstrasse 124, 4070 Basel, Switzerland; 2 F. Hoffmann-La Roche Ltd, Welwyn Garden City, UK; 3 Paraxel International, Milan, Italy; 4 Department of Rheumatology and Immunology, Inselspital, University Hospital Bern, Bern, Switzerland; 5 Department of Rheumatology, USB - University Hospital Basel, Basel, Switzerland; 6 Medical Center Monbijou, Bern, Switzerland

**Keywords:** Giant cell arteritis, Intravenous, Tocilizumab, Pharmacokinetics, Pharmacodynamics, Safety

## Abstract

**Background:**

Subcutaneous tocilizumab (TCZ SC) is approved globally for giant cell arteritis (GCA). This phase Ib study investigated the pharmacokinetics, pharmacodynamics, safety, and exploratory efficacy of intravenous (IV) TCZ 6 and 7 mg/kg in patients with GCA. This study explored an IV dose resulting in a minimum exposure level within the range of effective trough concentrations achieved with TCZ SC dosing in GCA and not exceeding the exposure of the well-tolerated 8 mg/kg IV every 4 weeks (Q4W) in rheumatoid arthritis (RA).

**Methods:**

Patients with GCA who had received ≥ 5 doses of TCZ IV 8 mg/kg Q4W and achieved remission were enrolled. Patients received 5 doses of TCZ IV 7 mg/kg Q4W in period 1 and, if still in remission, 5 doses of 6 mg/kg Q4W in period 2. Pharmacokinetic endpoints were maximum concentration (*C*_max_), minimum concentration (*C*_trough_), area under the curve over a dosing interval (AUC_*τ*_), and mean concentration (*C*_mean_) of TCZ after the last dose of each period. Other endpoints included pharmacodynamic markers, safety, and exploratory efficacy.

**Results:**

In 24 patients, the median (range) age was 65.5 (57–90) years, and 62.5% were female. TCZ exposures (*C*_max_ and AUC_*τ*_) were 11.2% and 20.0% lower at the 6- than 7-mg/kg dose. The mean interleukin 6 (IL-6) serum concentrations were elevated at baseline and remained elevated, with slightly higher concentrations in period 1 than in period 2. The mean serum soluble IL-6 receptor concentrations were elevated at baseline and comparable between the 2 doses at steady state. C-reactive protein levels and most erythrocyte sedimentation rates were within normal ranges throughout the study. Overall, 22 patients (91.7%) had ≥ 1 adverse event, and 4 (16.7%) had a serious adverse event. No patients experienced a GCA flare, and all remained in remission throughout the study.

**Conclusions:**

Both doses of TCZ IV Q4W were generally well tolerated in patients with GCA. The *C*_max_ and *C*_mean_ achieved with 6 mg/kg IV Q4W in patients with GCA were similar to those in patients with RA treated with 8 mg/kg IV Q4W, and *C*_trough_ was within the range observed in patients with GCA treated with SC dosing every week or every 2 weeks.

**Trial registration:**

ClinicalTrials.gov, NCT03923738

## Background

Giant cell arteritis (GCA), an immune-mediated vasculitis characterized by granulomatous inflammation affecting the medium and large arteries [1], is the most common primary systemic vasculitis and typically affects patients of Northern European ancestry ≥ 50 years of age [2–4]. Clinical manifestations include vision loss, headache, scalp tenderness, and jaw claudication. Noncranial symptoms, such as polymyalgia rheumatica (PMR) and limb claudication, may also occur [5]. When left untreated, GCA is associated with significant morbidity and severe complications, including blindness, aortic aneurysm, and stroke [6].

Glucocorticoids had been the mainstay of treatment for GCA until recently [7], and although they are highly effective at inducing remission and preventing acute damage (e.g., blindness), not all patients respond adequately to glucocorticoids alone [8–10], and up to 85% of patients experience an adverse event (AE) associated with their use [11, 12]. Moreover, tapering or discontinuation of glucocorticoids can lead to relapse of GCA symptoms [13–15]. Of the adjunctive treatments evaluated, there is limited evidence for glucocorticoid-sparing effects of methotrexate in part due to the heterogeneity of results between studies [16]. Tocilizumab (TCZ) has shown significant glucocorticoid-sparing effects in patients with GCA [10, 15, 17]. TCZ is a monoclonal antibody directed against the interleukin 6 (IL-6) receptor that inhibits signaling by the pro-inflammatory cytokine IL-6. A phase II investigator-initiated trial showed the efficacy of intravenous TCZ (TCZ IV) in the induction and maintenance of remission in patients with GCA [10]. Subsequently, a larger phase III study demonstrated the safety and efficacy of subcutaneous TCZ (TCZ SC) for the treatment of GCA [9], which led to the approval of TCZ SC globally for the treatment of GCA and its inclusion in multiple treatment recommendations [6, 18, 19].

Despite the benefit of TCZ related to sustained remission and glucocorticoid sparing in patients with GCA [17], TCZ SC is not accessible for some patients in the USA due to a gap in Medicare Part D prescription drug coverage. Furthermore, some patients, particularly older patients, have difficulty self-administering SC injections and/or adhering to a regimen of SC injections. Together, these considerations indicate an unmet medical need for alternate routes of TCZ administration in GCA; TCZ IV would provide a valuable treatment option by addressing both the access issue and the self-administration and/or adherence challenges some patients experience with SC treatment.

A positive benefit-risk profile of TCZ IV 8 mg/kg every 4 weeks (Q4W) in GCA was shown in the phase II, investigator-initiated, randomized controlled trial of 30 patients [10]. However, pharmacokinetic (PK) data were limited, and although the minimum (trough) concentrations (*C*_trough_) were within the therapeutic range established in the randomized trial of TCZ SC 162 mg every week (QW) or every 2 weeks (Q2W) [9], model-based predictions showed that average exposures (maximum concentration [*C*_max_] and area under the curve over a dosing interval [AUC_*τ*_]) at steady state were higher than those observed in the rheumatoid arthritis (RA) population treated with TCZ IV 8 mg/kg Q4W (data on file).

This phase Ib, open-label, dose-ranging study evaluated the PK, pharmacodynamics (PD), safety, and exploratory efficacy of TCZ 6 and 7 mg/kg administered by IV infusion Q4W in patients with GCA. The purpose was to identify the optimal TCZ IV dosing regimen in GCA, that is, a dosing regimen providing a minimum exposure level within the range of effective trough concentrations achieved with TCZ SC dosing in GCA and a maximum exposure not exceeding that of the well-tolerated 8-mg/kg IV Q4W dose in RA.

## Methods

### Study design

**Fig. 1:**
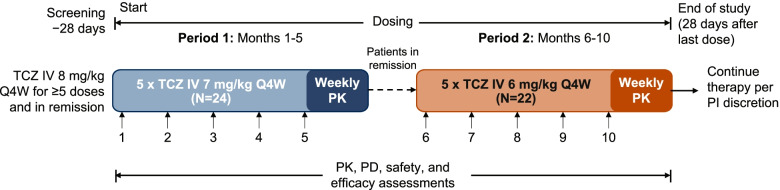
Study design. IV, intravenous; PD, pharmacodynamics; PI, principal investigator; PK, pharmacokinetics; Q4W, every 4 weeks; TCZ, tocilizumab

This phase Ib, open-label, dose-ranging study (NCT03923738) was divided into 2 periods (Fig. 1). In period 1, patients with GCA in remission received 5 consecutive doses of TCZ IV 7 mg/kg Q4W. Patients who were still in remission at the end of period 1 entered period 2 and received 5 consecutive doses of TCZ IV 6 mg/kg Q4W. A sixth dose could have been given in either period to accommodate patient availability for the intensive PK sampling during the last dosing cycle. Glucocorticoid use during the study was at the investigator’s discretion. The study was conducted in accordance with the International Council for Harmonisation E6 Guideline for Good Clinical Practice and the Declaration of Helsinki or Swiss regulations, whichever afforded greater patient protection. The protocol was approved by the ethics committee of the participating institution (Ethikkommission Nordwest- und Zentralschweiz, Basel, and Kantonale Ethikkommission Bern KEK, Bern).

### Patients

Patients with GCA who had received ≥ 5 consecutive doses of TCZ IV 8 mg/kg Q4W (off label) in clinical practice and had achieved remission (defined as the absence of flare and normalization of C-reactive protein [CRP] level [< 10 mg/L]) at the time of enrollment were enrolled in the study. Diagnosis of GCA was based on the following criteria: 1) age ≥ 50 years; 2) history of erythrocyte sedimentation rate (ESR) ≥ 50 mm/h or CRP level ≥ 24.5 mg/L if ESR was unavailable; 3) either unequivocal cranial symptoms of GCA (new-onset localized headache, scalp tenderness, temporal artery tenderness or decreased pulsation, ischemia-related vision loss, or otherwise unexplained mouth or jaw pain upon mastication) or symptoms of PMR (defined as shoulder and/or hip girdle pain associated with inflammatory morning stiffness); and 4) either temporal artery biopsy revealing features of GCA or evidence of large vessel vasculitis by angiography or cross-sectional imaging study such as magnetic resonance angiography, computed tomography angiography, or positron emission tomography-computed tomography. All patients gave written informed consent before participation in any study procedures.

### Safety and tolerability

Safety and tolerability were assessed by monitoring vital signs, clinical laboratory tests, and AEs. Patients were questioned about any AEs that they experienced, and events were also reported by patients spontaneously. The severity of AEs was determined according to the National Cancer Institute Common Terminology Criteria for Adverse Events, version 5.0 (NCI CTCAE v5.0). Cumulative incidence of AEs and person-year event rates (number of events divided by the sum of person-years of study duration) were computed, together with 95% confidence intervals based on the Poisson distribution of the event rate. Because patients had previously received TCZ, only event-driven immunogenicity assessments were performed in case of hypersensitivity reaction.

### Efficacy

Exploratory efficacy was assessed by the proportion of patients who experienced a flare, defined as the recurrence of signs or symptoms of GCA and/or ESR ≥ 30 mm/h attributable to GCA as determined by the investigator, and the proportion of patients in remission.

### Sample collection and analysis

Blood samples for the measurement of TCZ serum concentrations were collected before dosing and at the end of infusion on weeks 1, 8, 12, and 16 in both periods. Blood samples were also collected 1, 2, 3, and 4 weeks after the last dose in each period to estimate steady-state AUC. Blood samples for measurement of serum concentrations of IL-6 and soluble IL-6 receptor (sIL-6R) were collected at predose on weeks 1, 12, 16, and 20 of each period. Blood samples for measurement of CRP and ESR were collected on weeks 1, 4, 8, 12, 16, 17, 18, 19, and 20 of each period. Serum samples were analyzed for TCZ using a validated sandwich enzyme-linked immunoassay (ELISA). The lower limit of quantification was 100 ng/mL in the serum. The assay precision, as determined from the analysis control samples, was ≤ 8.7%. The accuracy ranged from 104.8 to 108.3%. IL-6 was quantified using 2 validated ELISA methods with different sensitivities. Calibration ranges were 3.12 to 300 pg/mL (low-sensitivity assay [LSA]) and 0.15 to 10.0 pg/mL (high-sensitivity assay [HSA]). The precision ranged from 6.3 to 14.6% (LSA) and from 0.8 to 5.1% (HSA), and the mean accuracy ranged from 93.4 to 100.1% (LSA) and from 91.0 to 94.3% (HSA). sIL-6R was quantified using a validated bridging ELISA method. The calibration range was 12.5 to 800 ng/mL. The coefficients of variation of quality control samples ranged from 5.9 to 7.2%, and the mean accuracy ranged from 86.9 to 96.1%. The serum samples were analyzed for TCZ, IL-6, and sIL-6R concentrations by QPS (QPS Netherlands B.V., Groningen, the Netherlands). Serum CRP was determined by the Roche Diagnostics Elecsys CRP assay. ESR was measured using the Westergren method by study coordinators and/or study nurses at the sites.

### Pharmacokinetics

The following TCZ PK parameters at steady state were calculated using noncompartmental methods (Phoenix® WinNonlin® 8.2, Pharsight Corporation, Certara USA, Princeton, NJ): *C*_max_, time to *C*_max_ (*T*_max_), *C*_trough_, AUC_*τ*_ over a dosing interval (*τ*), mean concentration (*C*_mean_) calculated as AUC_*τ*_/*τ*, and half-life (*T*_1/2_) of TCZ after the last dose of each period.

### Statistical methods

All PK and PD parameters were subjected to descriptive analyses, including arithmetic mean and standard deviation (SD) or range. Statistical analyses were performed using SAS, version 9.4 (SAS Institute, Cary, NC).

Based on the known PK variability of TCZ, a sample size of 17 patients was predicted to provide > 80% power to characterize the geometric mean estimate of the observed *C*_trough_ and *C*_max_ so that the 95% confidence interval would fall within 80 to 125% of the geometric mean estimate of the corresponding PK parameter. Approximately 25 patients were to be enrolled to account for potential study dropouts.

## Results

### Patient disposition and baseline characteristics

**Fig. 2:**
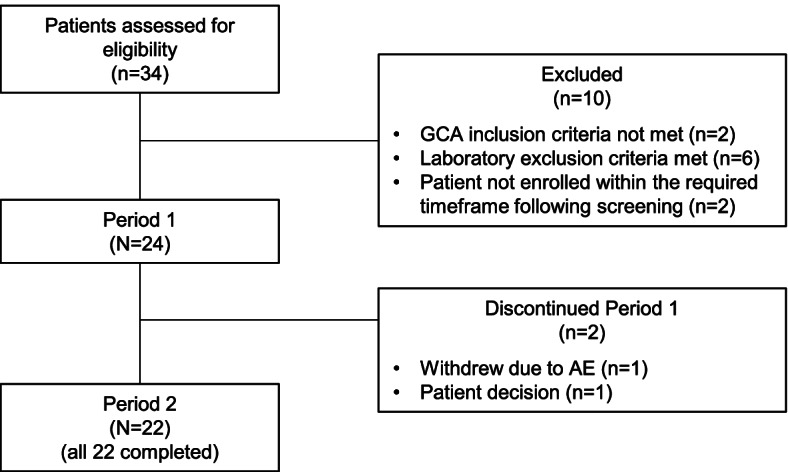
Patient disposition. AE, adverse event; GCA, giant cell arteritis

Between August 2019 and February 2020, 24 patients were enrolled (Fig. 2). All patients had a history of ESR ≥ 50 mm/h and/or CRP ≥ 24.5 mg/L at the time of GCA diagnosis (Table 1). Of the 24 patients enrolled, 15 (62.5%) were female and 9 (37.5%) were male, and all patients except one were White (Table 2). At baseline (day 1 of period 1), the median (range) age of patients was 65.5 (57–90) years. All 24 patients had received ≥ 5 consecutive doses of TCZ IV 8 mg/kg Q4W and were in clinical remission at baseline, with ESRs < 30 mm/h and CRP levels < 10 mg/L. The median (range) duration of GCA was 2.4 (0.8–13.2) years, and 7 patients (29.2%) reported glucocorticoid use (prednisone or prednisolone) for GCA, all of which were received orally and at doses of ≤ 5 mg per day.

**Table 1:** Giant cell arteritis disease characteristics at the time of diagnosis

***n*** (%)	All patients, ***N*** = 24
History of ESR ≥ 50 mm/h	17 (70.8)
History of CRP ≥ 24.5 mg/L	21 (87.5)
Localized headache	16 (66.7)
Scalp tenderness	6 (25.0)
Temporal artery tenderness	7 (29.2)
Temporal artery decreased pulsation	2 (8.3)
Ischemia-related vision loss	2 (8.3)
Otherwise unexplained mouth or jaw pain upon mastication	8 (33.3)
PMR symptoms	15 (62.5)
Temporal artery biopsy performed	15 (62.5)
Positive temporal artery biopsy results	13 (54.2)
Angiography or cross-sectional imaging performed	20 (83.3)
Magnetic resonance angiography	13 (65.0)
Positron emission tomography-computed tomography	6 (30.0)
Ultrasound	1 (5.0)
Large vessel vasculitis	18 (75.0)

*CRP* C-reactive protein, *ESR* Erythrocyte sedimentation rate, *PMR* Polymyalgia rheumatica

**Table 2:** Baseline^a^ demographics and disease characteristics

	All patients, ***N*** = 24
Sex, *n* (%)	
Female	15 (62.5)
Male	9 (37.5)
Age, median (range), years	65.5 (57–90)
Age group, *n* (%), years	
< 65	11 (45.8)
≥ 65	13 (54.2)
Race, *n* (%)^b^	
Asian	1 (4.2)
White	23 (95.8)
Weight, median (range), kg	69.5 (45–113)
BMI, median (range), kg/m^2^	25.3 (17.4–36.5)
Smoking history, *n* (%)	
Never	12 (50.0)
Current	4 (16.7)
Former	8 (33.3)
ESR, median (range), mm/h	4.0 (0–25)
CRP, median (range), mg/L	0.20 (0.20–5.81)
Duration of GCA, median (range), years	2.4 (0.8–13.2)
Glucocorticoid use for GCA, *n* (%)	7 (29.2)

*BMI* Body mass index, *CRP* C-reactive protein, *ESR* Erythrocyte sedimentation rate, *GCA* Giant cell arteritis

^a^Baseline is day 1 of period 1

^b^Self-reported

In period 1, 24 patients received TCZ IV 7 mg/kg Q4W, with a median treatment duration of 20.0 weeks and a median (range) of 5 (1-6) doses; 3 patients received a sixth dose. Two patients discontinued in period 1 (1 due to an AE and 1 due to a patient decision). In period 2, 22 patients received TCZ IV 6 mg/kg Q4W, with a median treatment duration of 20.0 weeks. All patients received 5 doses of 6 mg/kg, and no patients discontinued. The total patient-years of exposure to TCZ was 9.02 years in period 1 and 8.48 years in period 2.

### Pharmacokinetics

**Fig. 3:**
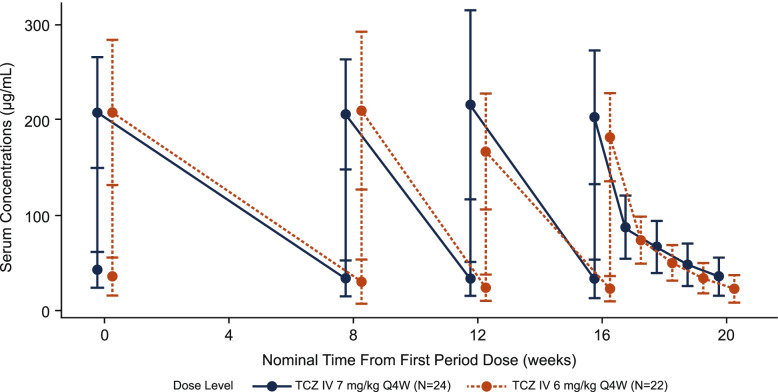
Arithmetic mean (SD) serum concentration of TCZ vs time profiles following TCZ IV 7 mg/kg Q4W in period 1 and TCZ IV 6 mg/kg Q4W in period 2, linear scale. Three patients received a sixth dose at week 20 in period 1. To align their end-of-period profile with those of the other patients, their week 16 data have been excluded from this plot. Only samples at predose and end of infusion were collected after the first, third, and fourth doses in each period. IV, intravenous; Q4W, every 4 weeks; SD, standard deviation; TCZ, tocilizumab

All 24 patients enrolled were included in the PK analysis, but only 22 provided steady-state PK parameters in both periods. During period 1, two samples at the end of the infusion of TCZ were collected from the same arm used for TCZ administration; these data were excluded from the descriptive summary statistics. The mean PK profile following TCZ IV 7 mg/kg Q4W in period 1 was of a similar shape to the mean PK profile following TCZ IV 6 mg/kg Q4W in period 2, with a slightly lower exposure at the 6-mg/kg dose level (Fig. 3). Following IV dosing of 7 and 6 mg/kg Q4W in patients with GCA, the observed median TCZ *C*_max_ was 197 and 178 μg/mL, respectively, and the median AUC_*τ*_ was 2130 and 1610 day•μg/mL at steady state (Table 3). Compared with the 7-mg/kg dose, TCZ exposures (*C*_max_ and AUC_*τ*_) were on average 11.2% and 20.0% lower with the 6-mg/kg dose. The median TCZ *C*_mean_ at steady state was 76.0 for the 7-mg/kg dose and 57.5 μg/mL for the 6-mg/kg dose, and the observed median *C*_trough_ levels were 37.2 and 22.7 μg/mL for the 7- and 6-mg/kg doses, respectively.

**Table 3:** Steady-state PK parameters of TCZ IV 7 and 6 mg/kg Q4W

PK parameters, mean, median (range)	7 mg/kg IV (period 1), ***n*** = 22^a^	6 mg/kg IV (period 2), ***n*** = 22
*C*_max_, μg/mL	205	182
	197 (118–352)	178 (115–320)
AUC_*τ*_, day•μg/mL	2150	1720
	2130 (1120–4300)	1610 (921–3070)
*C*_mean_, μg/mL	76.9	61.5
	76.0 (40.1–154)	57.5 (32.9–110)
*C*_trough_, μg/mL	35.3	22.7
	37.2 (6.59–69.0)	22.7 (3.38–54.5)
*T*_max_, days	0.38	0.05
	0.05 (0.04–6.97)	0.05 (0.04–0.06)
*T*_1/2_, days^b^	19.0	12.1
	14.8 (5.86–120.0)	13.2 (4.69–21.9)

*AUC*_*τ*_ Area under the curve over a dosing interval (*τ*), *C*_*max*_ Maximum concentration, *C*_*mean*_ Mean concentration (AUC_*τ*_/*τ*), *C*_*trough*_ Minimum (trough) concentration, *IV* Intravenous, *PK* Pharmacokinetics, *Q4W* Every 4 weeks, *T*_*1/2*_ Half-life, *TCZ* Tocilizumab, *T*_*max*_ Time to *C*_max_

^a^*n* = 21 for *C*_max_

^b^*T*_1/2_ of TCZ is concentration-dependent; extrapolation from noncompartmental analysis should be made with caution

### Pharmacodynamics

**Fig. 4:**
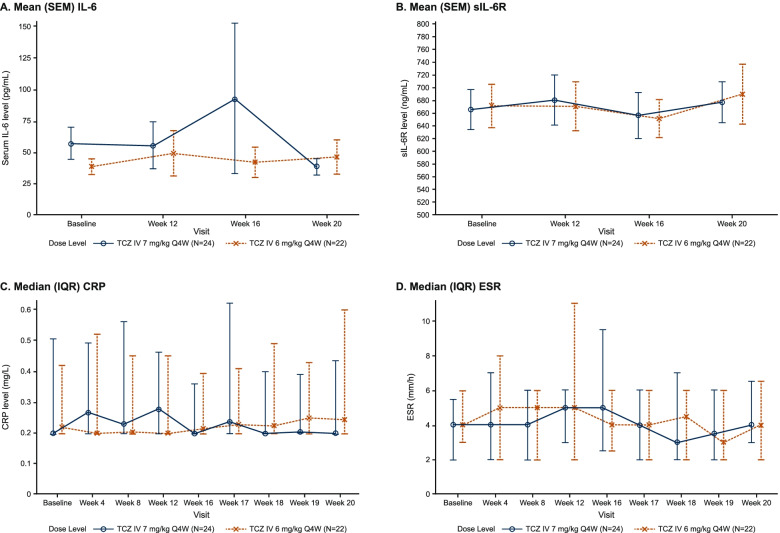
Serum concentrations of IL-6 (**A**), sIL-6R (**B**), and CRP (**C**) vs time profiles and ESR over time (**D**) by visits and dose level. Baseline was defined as the last nonmissing assessment on or before the first TCZ dose of the dosing period. The last dose could be the fifth or sixth dose, according to the investigator’s decision. Three patients received a sixth dose at week 20 in period 1. To align their end-of-period profile with those of the other patients, their week 16 data have been excluded from this plot. ULN for CRP was 10 mg/L, and for ESR, it was 30 mm/h. CRP, C-reactive protein; ESR, erythrocyte sedimentation rate; IL-6, interleukin 6; IQR, interquartile range; IV, intravenous; PK, pharmacokinetics; Q4W, every 4 weeks; SEM, standard error of the mean; sIL-6R, soluble interleukin 6 receptor; TCZ, tocilizumab; ULN, upper limit of normal

The mean IL-6 serum concentrations were elevated at baseline as expected due to recent TCZ treatment, with numerically higher concentrations in period 1 (mean [SD], 57.80 [61.15] pg/mL) than in period 2 (mean [SD], 39.46 [25.99] pg/mL) (Fig. 4A). The IL-6 serum concentrations remained almost stable throughout the study following TCZ IV 7 mg/kg Q4W in period 1 and TCZ IV 6 mg/kg Q4W in period 2 except at week 16 for the 7-mg/kg dose level, which was driven by the elevated IL-6 serum concentration of 1 patient; the cause of the elevation was not identified. The mean sIL-6R concentrations were elevated at baseline as expected due to recent TCZ treatment (mean [SD], 665.8 [153.81] ng/mL and 671.3 [152.69] ng/mL for 7 and 6 mg/kg, respectively) and comparable between the 2 doses at steady state (Fig. 4B). CRP levels and most ESRs were within normal ranges at baseline, as expected for patients in remission, and remained normalized (or controlled) throughout the study (Fig. 4C, D).

### Safety

Overall, 22 patients (91.7%) had ≥ 1 AE (19 patients [79.2%] in period 1 [7 mg/kg] and 9 [40.9%] in period 2 [6 mg/kg]; Table 4). Infections and infestations were the most frequently reported AE by System Organ Class (13 patients [54.2%] in period 1 and 6 [27.3%] in period 2). Two patients (8.3%) experienced a grade ≥ 3 AE. The overall rate of AEs in periods 1 and 2 were 388.0 events per 100 person-years (95% CI, 270.3 to 539.7) and 188.7 events per 100 person-years (95% CI, 107.8 to 306.4), respectively. The majority of AEs (70.8%) were not TCZ-related. One patient in period 1 experienced an AE (nonserious grade 3 AE of postoperative thrombocytopenia) that led to the withdrawal of treatment but was considered unrelated to study treatment. Overall, 4 patients (16.7%) reported a serious adverse event (SAE; pneumococcal pneumonia, aortic aneurysm rupture, and lower gastrointestinal hemorrhage [no diverticulitis observed; event assessed by the investigator as related to anticoagulation medication] in period 1 and positional vertigo in period 2). Only the pneumococcal pneumonia event in a patient not receiving concomitant glucocorticoids was considered TCZ-related by the investigator. Three of the SAEs (pneumococcal pneumonia, aortic aneurysm rupture, and lower gastrointestinal hemorrhage) led to treatment interruption; 1 patient (aortic aneurysm rupture) ultimately withdrew in period 1 due to previously noted postoperative thrombocytopenia, and 2 patients continued and completed the study after treatment delay at week 4. There were no deaths during the study.

**Table 4:** Overview of AEs

	7 mg/kg IV (period 1), ***n*** = 24	6 mg/kg IV (period 2), ***n*** = 22	All patients (periods 1 and 2), ***N*** = 24
Patients with ≥ 1 AE, *n* (%)	19 (79.2)	9 (40.9)	22 (91.7)
Total no. of AEs, *n*	35	16	51
Total no. of deaths, *n*	0	0	0
Total no. of patients with ≥ 1 AE, *n* (%)			
Leading to withdrawal from treatment	1 (4.2)	0	1 (4.2)
Leading to dose modification or interruption	3 (12.5)	0	3 (12.5)
Grade ≥ 3^a^	2 (8.3)	0	2 (8.3)
Treatment related^b^	6 (25.0)	1 (4.5)	7 (29.2)
Patients with ≥ 1 SAE, *n* (%)	3 (12.5)	1 (4.5)	4 (16.7)
Total no. of patients with ≥ 1 SAE, *n* (%)			
Leading to withdrawal from treatment	0	0	0
Leading to dose modification or interruption	3 (12.5)	0	3 (12.5)
Treatment related^b^	1 (4.2)	0	1 (4.2)
Total no. of patients with selected AEs, *n* (%)			
Infections	13 (54.2)	6 (27.3)	16 (66.7)
Neutropenia	0	0	0
Thrombocytopenia	1 (4.2)	0	1 (4.2)
Total no. of patients with an AE of special interest, *n* (%)			
Serious bleeding events	2 (8.3)	0	2 (8.3)
Serious infections	1 (4.2)	0	1 (4.2)
Anaphylactic reactions	0	0	0
Demyelinating disorders	0	0	0
Gastrointestinal perforations	0	0	0
Hypersensitivity reactions	0	0	0
Malignancies	0	0	0
Myocardial infarctions	0	0	0
Opportunistic infections	0	0	0
Serious hepatic events	0	0	0
Stroke	0	0	0

Investigator text for AEs encoded using MedDRA, version 23.1. Multiple occurrences of the same AE in 1 individual are counted only once except for the “Total no. of AEs” row, in which multiple occurrences of the same AE are counted separately

*AE* Adverse event, *IV* Intravenous, *MedDRA* Medical Dictionary for Regulatory Activities, *SAE* Serious adverse event

^a^Incidence and severity of adverse events as determined by the National Cancer Institute Common Terminology Criteria for Adverse Events, version 5.0, were evaluated

^b^As determined by the investigator

Hematology, hepatic, and lipid laboratory abnormalities observed during the study were consistent with the known TCZ safety profile. All low absolute neutrophil count abnormalities were either grade 1 or 2. All platelet count decreases were grade 1 except for 1 patient with grade 3 postoperative thrombocytopenia, which was reported as unrelated to the study treatment by the investigator. No grade ≥ 2 high alanine aminotransferase, aspartate aminotransferase, or total bilirubin abnormalities were reported during the study, and no Hy’s law cases were reported.

### Exploratory efficacy

No patients experienced a GCA flare or any signs or symptoms of GCA, and all patients remained in remission throughout the study.

## Discussion

In this phase Ib study of patients with GCA who were in remission after receiving TCZ IV 8 mg/kg Q4W for ≥ 5 consecutive doses and subsequently received 2 dose levels of TCZ IV, the mean PK profile following TCZ IV 7 mg/kg Q4W in period 1 was of a similar shape to the mean PK profile following TCZ IV 6 mg/kg Q4W in period 2, with a lower exposure at the 6-mg/kg dose level. These study results support a dose of TCZ IV 6 mg/kg Q4W to maintain remission in patients with GCA.

The minimum exposure levels (*C*_trough_) of the 7- and 6-mg/kg IV dose were within the range of effective trough concentrations achieved with 162 mg SC QW and Q2W in patients with GCA (median [range], 67.2 [10.7–145] and 7.7 [0.1–37.3], respectively) [9]. The maximum exposure results of the TCZ IV 6-mg/kg Q4W dose were similar to the safe and well-tolerated exposure seen with 8 mg/kg IV Q4W in patients with RA (median [range], AUC_*τ*_, 1512 [476–7283] day•μg/mL [data on file]; *C*_mean_, 54.0 [17.0–260] μg/mL; and *C*_max_, 176 [75.4–557] μg/mL) [20] (Table 5). The maximum exposure results (AUC_*τ*_, *C*_mean_, and *C*_max_) of the TCZ IV 7-mg/kg Q4W dose exceeded these values. Based on population PK modeling, using the model initially developed for patients with RA [21], patients with GCA appear to have a lower linear apparent clearance than patients with RA, which results in a 50% difference between the predicted steady-state exposures in the 2 populations. The reason for the difference between patients with GCA and those with RA is suspected to be disease-specific; however, the exact reason remains unknown. None of the covariates examined (e.g., age, sex, body weight) in the present study and previous studies in GCA [9, 10] were shown to explain the differences.

**Table 5:** Summary of pharmacokinetic steady-state TCZ exposure parameters

Studies	Dosing regimen	Patient population	Number	AUC_***τ***_ (day•μg/mL)	***C***_**mean**_ (μg/mL)	***C***_**max**_ (μg/mL)	***C***_**trough**_ (μg/mL)
Present study^a^	6 mg/kg IV Q4W	GCA	22	1610 (921–3070)	57.5 (32.9–110)	178 (115–320)	22.7 (3.38–54.5)
Present study^a^	7 mg/kg IV Q4W	GCA	22	2130 (1120–4300)	76.0 (40.1–154)	197 (118–352)	37.2 (6.59–69.0)
Phase II IIS (NCT01450137) [10]^b^	8 mg/kg IV Q4W	GCA	20	2249 (457–5778)	80.3 (16–206)	190 (48.5–538)	35.5 (0–145)
GiACTA (NCT01791153) [9]^b^	162 mg SC QW	GCA	100	495 (82–1042)	70.6 (11.7–149)	72.1 (12.2–151)	67.2 (10.7–145)
GiACTA (NCT01791153) [9]^b^	162 mg SC Q2W	GCA	49	191 (97.7–686)	13.7 (0.5–49)	17.2 (1.1–56.2)	7.7 (0.1–37.3)
PopPK RA^c^	8 mg/kg IV Q4W	RA	2155	1512 (476–7283)	54.0 (17.0–260)	176 (75.4–557)	13.4 (0.1–154)

Values are median (range)

*AUC*_*τ*_ Area under the curve over a dosing interval (*τ*), *C*_*max*_ Maximum concentration, *C*_*mean*_ Mean concentration (AUC_*τ*_/*τ*), *C*_*trough*_ Minimum (trough) concentration, *GCA* Giant cell arteritis, *IIS* Investigator-initiated study, *IV* Intravenous, *PopPK* Population PK, *PK* Pharmacokinetic, *QW* Every week, *Q2W* Every 2 weeks, *Q4W* Every 4 weeks, *RA* Rheumatoid arthritis, *SC* Subcutaneous, *TCZ* Tocilizumab

^a^Noncompartmental analysis

^b^PopPK analysis

^c^PopPK analysis of RA studies WA17822 (NCT00106548), WA17824 (NCT00109408), WA18062 (NCT00106522), WA18063 (NCT00106574), and WA22762 and NA25220 (NCT01662063) (data on file)

IL-6 serum concentrations were relatively high at baseline (≈ 50 pg/mL) because patients received ≥ 5 consecutive TCZ doses before entering the study (IL-6 receptor blockade by TCZ inhibits IL-6 elimination, which is principally receptor-mediated) [22]. IL-6 levels remained elevated throughout the study, reflecting an equilibrium between its formation and its slower clearance due to IL-6 receptor blocked by TCZ. Likewise, sIL-6R levels also remained elevated, reflecting the slower clearance of the TCZ-receptor complex relative to the native substrate-receptor complex.

Treatment with TCZ was generally well-tolerated, and no new safety concerns were identified. The AEs observed during period 1 (TCZ IV 7 mg/kg Q4W) and period 2 (TCZ IV 6 mg/kg Q4W) were consistent with AEs observed in other TCZ GCA studies [9, 10], the large clinical trial data set from RA, and the established safety profile of TCZ. The numerically higher incidence and rate of AEs in period 1 of this study should be interpreted with caution due to the small sample size and, for the rate, the fact that 2 patients were withdrawn from the study during period 1, one of whom contributed the highest number of AEs/SAEs (6 events) in period 1. Infections are a concern in patients with GCA due to age and concomitant glucocorticoid treatment, and a higher rate of severe infection has been seen in older patients with GCA than in those with RA, as reported in an analysis of clinical trial and claims data [23]. During the present study, 1 serious infection (pneumococcal pneumonia) was reported; however, the overall number of patients was low, and the follow-up time was limited.

Patients began the present study in remission (after initial dosing with TCZ) and stayed in remission with 7- and 6-mg/kg dosing in both treatment periods, and no patients experienced a GCA flare. Notably, only 7 and 2 patients were receiving glucocorticoids in period 1 and 2, respectively, all at a prednisone-equivalent dose of ≤ 5 mg per day, which supports the glucocorticoid-sparing effect of TCZ. Doses of TCZ IV < 8 mg/kg have also demonstrated effectiveness in several real-world observational studies of patients with GCA. TCZ IV at a dose of 4 mg/kg Q4W effectively induced and maintained remission in 11 of 13 (85%) older patients (median age, 78 years) with GCA and often severe comorbidities [24]. In another small retrospective study, a gradual dose reduction of TCZ IV from 8 to 4 mg/kg, along with an increased dosing interval, in patients with GCA who were in remission and receiving long-term treatment with TCZ was effective for maintaining sustained remission in 12 of 13 patients (92%) [25]. While these lower doses of TCZ were effective in treating GCA (i.e., inducing and maintaining remission) in these small observational studies, TCZ doses lower than the approved dosage may carry the risk of reduced efficacy, possibly leading to vision loss and other ischemic complications. As previously mentioned, the dosing regimen of TCZ IV 6 mg/kg Q4W provides trough concentrations similar to effective trough concentrations achieved with SC dosing regimens in patients with GCA. A dosing regimen of TCZ IV 6 mg/kg Q4W is expected to be effective in the treatment of GCA based on the maintenance of remission and the similarity of exposure.

In the phase III TCZ SC GCA trial [9] and the phase II TCZ IV GCA trial [10], the duration of treatment with TCZ was 1 year, but the ideal length of treatment with TCZ for GCA is unknown. Observational studies have shown that patients can maintain remission without continued TCZ or glucocorticoid treatment; however, in patients who achieved remission with TCZ, approximately 50 to 60% relapsed after TCZ was discontinued [15, 26, 27]. Recommendations for TCZ treatment duration vary from deciding the length of treatment on an individual basis [6] to discontinuation after 1 year [18]. The duration of TCZ treatment should be carefully discussed in shared decision-making between healthcare providers and patients and consider patient factors such as comorbidities, type of GCA manifestations, and risk of GCA relapse and glucocorticoid-related AEs. In patients who relapse after discontinuation of TCZ, retreatment, with and without glucocorticoids, has been shown to be effective at restoring remission [15].

### Limitations

Per the study design, patients entered this study in remission after receiving ≥ 5 doses of TCZ IV 8 mg/kg Q4W. This study was open-label; however, the PK and PD endpoints were not expected to be affected by dose awareness. The small sample size should be taken into consideration when interpreting the safety and exploratory efficacy data.

## Conclusions

Both dose levels of TCZ IV (6 and 7 mg/kg) Q4W were generally well tolerated in patients with GCA, and patients stayed in remission throughout the study. The *C*_max_ and *C*_mean_ achieved with 6 mg/kg IV Q4W in patients with GCA were similar to those seen in patients with RA treated with 8 mg/kg IV Q4W, and the *C*_trough_ was within the range observed in patients with GCA treated with 162 mg SC QW and Q2W. These study results support a dose of TCZ IV 6 mg/kg Q4W in patients with GCA.

## Data Availability

Given the small study population the decision to share the patient level data needs to be handled on a case by case basis to determine if the clinical data can be adequately anonymized to give an acceptably low risk of patient-re identification. Qualified researchers may submit an enquiry through the data request platform, Vivli, at https://vivli.org/ourmember/roche/, however this does not guarantee that the data can be shared. For up to date details on Roche's Global Policy on the Sharing of Clinical Information and how to request access to related clinical study documents, see: go.roche.com/data_sharing Anonymized records for individual patients across more than one data source external to Roche can not, and should not, be linked due to a potential increase in risk of patient re-identification.

## Abbreviations

AE: Adverse event
AUC: Area under the curve
AUC_*τ*_: Area under the curve over a dosing interval
*C*_max_: Maximum concentration
*C*_mean_: Mean concentration
CRP: C-reactive protein
*C*_trough_: Minimum (trough) concentration
ELISA: Enzyme-linked immunoassay
ESR: Erythrocyte sedimentation rate
GCA: Giant cell arteritis
HSA: High-sensitivity analysis
IL-6: Interleukin 6
IV: Intravenous
LSA: Low-sensitivity assay
PD: Pharmacodynamic
PK: Pharmacokinetic
PMR: Polymyalgia rheumatica
QW: Every week
Q2W: Every 2 weeks
Q4W: Every 4 weeks
RA: Rheumatoid arthritis
SAE: Serious adverse event
SC: Subcutaneous
SD: Standard deviation
sIL-6R: Soluble interleukin 6 receptor
*T*_1/2_: Half-life
TCZ: Tocilizumab
*T*_max_: Time to maximum concentration
